# The Diagnosis and Treatment of Destructive Diseases of the Dental Pulp; and a Method of Removing the Dead Tissue from the Canals*Read before the Chicago Dental Society, October 16, 1917, and as published in the *Dental Review* for December, 1917.

**Published:** 1917-12

**Authors:** J. P. Buckley

**Affiliations:** Chicago


### THE DIAGNOSIS AND TREATMENT OF DESTRUCTIVE DISEASES OF THE DENTAL PULP; AND A METHOD OF REMOVING THE DEAD TISSUE FROM THE CANALS *

BY J. P. BUCKLEY, PH.G., D.D.S. CHICAGO

It is often a difficult problem for the conscientious operator to decide upon a method of procedure that will best conserve the interest of the patient. Especially is this true when we are trying to determine whether an exposed pulp should be treated and saved or whether it would be best for all concerned to destroy and remove it. It is so easy in most cases to anesthetize or devitalize the pulp that many dentists resort to this method of treatment in nearly all of the diseases of the organ. When we consider the fine and tortuous canals which we so frequently encounter and the difficulty both of removing the dead tissue from such canals and of thoroughly filling them subsequently, it is a question whether or not the best interest of the patient is conserved by adopting this method as the general practice.

While it is true, generally speaking, that pulps which are diseased beyond the condition of active hyperemia should be removed, it is also true that this organ has been destroyed far too ruthlessly in the past. The work of Rosenow, the observations of Hunter, Murphy, Billings, the Mayos, Crile and others of the medical profession, and the investigations of Grieve, Rhein, Gilmer, Price, Hartzell, Logan, Moorehead, Black and others of our own profession, along the line of periapical infections and their systematic results, should teach us to conserve the dental pulp in every case where it is practicable and consistent with good dentistry. Thousands upon thousands of normal

* Read before the Chicago Dental Society, October 16, 1917, and as published in the *Dental Review* for December, 1917.

pulps have been sacrificed for no other reason than that the dentin of the tooth was hypersensitive. When we think of the difficulty—not to mention the carelessness—under which these pulps are often removed, and of the frequent periapical infection resulting therefrom, we behold a sad spectacle, indeed! Such results should teach us that this practice must cease.

I have never heretofore, nor do I intend to now, to plead for the life of the pulp when the reading of the clinical symptoms clearly indicates its removal; but I do mean to say that in all cases an effort should be made to determine as nearly as possible the exact pathological condition, and our decision should be based upon these findings. From the viewpoint of the diagnostician there are few subjects of greater importance than those of hyperemia and inflammation of the dental pulp. In the treatment of the dental pulp, as in all other therapy, it is highly essential that we differentiate between the various kinds of diseases of this organ, and by means of reasoning, using common sense and judgment, and the process of exclusion, arrive at a correct diagnosis before applying our therapeutics.

### THE NORMAL DENTAL PULP

To know a tissue or organ in disease one must first know it in health. Therefore, let us briefly review the anatomy and physiology of the dental pulp. Our histologists inform us that this organ is the remains of the original dental papilla, changed somewhat to meet its present environment, and is composed of soft embryonal connective tissue with an outer layer of odontoblastic cells, the whole being well supplied with blood vessels and nerves. The blood vessels of the pulp are supposed to communicate with the general circulation through the apical foramen or foramina of the tooth. One or more small arterial trunks enter the pulp cavity at the apex, and, coursing occlusally through the center of the tissue, give off many branches. Near the occlusal end of the pulp they further divide into capillaries, and form a fine plexus around the peri-phereal portion of the pulp. The blood vessels are generously distributed through the tissue. The veins form a similar plexus, and a central vein, analogous to the artery, receives the blood from these many venules and conducts it through the apical foramen. The nerves of the pulp are transmitted through the apical foramen together with the blood vessels. Several bundles of medullated nerve fibers enter the foramen and break up into a plexus of nerves, which are widely distributed through the pulp tissue. (Turner.)

### CHARACTER OF BLOOD VESSELS

The walls of the pulpal blood vessel are unusually thin. The arteries are found to be almost devoid of the external fibrous coat; and, according to Noyes, the muscular layer is represented by a single involuntary fiber, while the walls of the veins are formed by a single layer of endothelial cells. It has long been known that the pulp tissue, like the brain, contains no lymphatics, and when we recall the thinness of the walls of the blood vessels and that organ is confined within a cavity having unyielding walls, the absence of lymphatics becomes of the greatest pathologic significance.[1]

### DIAGNOSIS OF ACTIVE AND PASSIVE HYPEREMIA, AND TRUE PULPITIS

In the pulp tissue, as in all living tissues of the body, we may have two kinds of hyperemia—active, or arterial, and passive, or venous. The former is defined as an excessive amount of blood in the arteries and the latter as an excessive amount of blood in the veins. Active hyper-

[1] Noyes has recently been led to believe from investigation that lymphatics are present in the pulp tissue.

emia is due to a determination of blood to a given part as the result of reaction to an irritant, and as long as it remains without an excessive immigration of the white blood corpuscles, it is active, or arterial hyperemia. The cause of passive hyperemia is different. Here the blood may be held back by some obstruction in the veins which prevents the return of blood to the heart. Inglis says: "The backing up of the blood following venous obstruction produces tension upon the vessels followed by diapedesis of red corpuscles and exudation of watery fluid—the condition of edema. In inflammation arterial hyperemia appears as the first stage, followed by a collection of leucocytes along the walls of the small veins, and the immigration of some into the perivascular tissue. As this eventually leads to stoppage of the blood current, and even to stasis, the condition is essentially a venous hyperemia. Accompanying the immigration of leucocytes is an exudation of lymph, highly coagulable in character, which distends the lymph spaces in the tissue and produces the characteristic swelling."

The thing of special interest here is that in inflammation or even in venous hyperemia certain blood elements escape through the temporarily dilated vessels into the perivascular tissue, which becomes coagulated, and when the tissue involved is the dental pulp, which, as we have learned is without lymphatics, this coagulation of the fluid elements of the blood means death. The procedure of many practitioners when an aching tooth presents involving the pulp is simply to find out whether the latter is dead or alive. If alive, cocain hydrochlorid or arsenic trioxid is generally applied, or else the tooth is treated tentatively by placing cotton dipped in oil of cloves, or some other soothing remedy in the cavity, and the patient is dismissed without making any effort to ascertain the condition of the pulp—whether it is in the stage of active hyperemia or passive hyperemia or true inflammation. If, on the other hand, the pulp is found to be dead, the tooth is too frequently treated by opening up the pulp chamber and placing therein a pledget of cotton dipped into the most convenient remedy at hand, leaving the cavity unsealed for fear of causing septic pericemenitis or an acute alveolar abscess, and making no further effort to ascertain whether the pulp has been infected by pyogenic microorganisms, producing pus on the exposed surface, or, as sometimes occurs, within the substance of the tissue, or whether the germs inaugurating the changes are of the saprophitic variety causing pulp decomposition with gaseous end products. It is highly essential, therefore, when a patient presents with an aching tooth, that we take the time and trouble to determine not only whether we have a live or a dead pulp with which to contend, but, if the pulp is alive, whether the condition is one of active hyperemia only or passive hyperemia or inflammation; or, if the pulp is dead, whether the condition is a so-called septic pulp with pus formation (pulpal abscess) or one of true gangrene with gaseous end products. To confirm our diagnosis the tooth should be isolated and kept dry, preferably with the rubber dam, and a careful examination made. On no other basis can rational therapeutics be practiced.

### DIFFERENTIAL DIAGNOSIS

It is generally a simple matter, by asking a few questions and observing conditions, to differentiate between the pathologic condition of a vital pulp sufficiently to determine whether the organ had better be saved or destroyed. In acute active hyperemia the pain occurs only when the irritant is applied, and subsides almost momentarily without treatment. Logan states that "active hyperemia exists in a pulp when the pain begins with a known irritation, lasting only a few moments, or minutes at the most, and subsiding without treatment and not starting again until the application of another known irritant is made." Such irritants may be heat, cold, impaction of food, etc.

### TREATMENT OF ACTIVE HYPEREMIA

The treatment of this condition consists in protecting the tooth from the irritant which caused the disease. If a cavity exists which is causing the trouble an anodyne remedy is indicated. One treatment with phenol compound, or other remedies possessing similar properties, should cure a case of active hyperemia due to caries of the tooth. At the second sitting, if the case has a favorable history, the rubber dam should be adjusted and the dressing and carious dentin removed, after which a base of cement may be inserted, the cavity prepared, and the tooth filled. In those cases where the cavity is deep and encroaches upon the pulp, and where the removal of the softened dentin practically exposes the organ, it is necessary, in order to save the pulp, to protect it by the use of some antiseptic paste. Here I use thymolized calcium phosphate as the power and oil of cloves as the liquid to make such a paste. Just a little aristol or europhen (iodin compounds) is added to the paste after it is mixed to the proper consistency, which should be like softened gelatin. In cases of actual exposure, the pulp should be destroyed and removed, unless some exceptional reason obtains for preserving the life and functuating powers of the organ; and even in cases of near exposure, one must be careful that the pulp is acutely alive and normal, for in many cases it simply dies without the manifestation of the usual pathologic symptoms. Such stimuli as heat, cold and electricity may be used to test the acuteness of the vitality of the pulp. Should it fail to respond to these tests, or should it ache severely without ceasing momentarily, it had better be removed.

### TREATMENT OF PASSIVE HYPEREMIA AND TRUE INFLAMMATION

As far as therapeutics is concerned, it is of little importance to know whether the pathologic condition of the pulp is passive hyperemia or true inflammation. In either case we have transudation of the fluid elements of the blood and diapedesis of the red corpuscles occurring, and this means death ultimately. In passive hyperemia the pain is more constant than in active hyperemia, and may start without the application of a known irritant. In inflammation the pain is continuous and of a boring character, or it may be lancinating, and is frequently described by the patient as a "jumping toothache." When the reading of the symptoms indicates either of these conditions, our therapeutics differs from that prescribed for active hyperemia, for here, as has been previously stated, the pulp must be destroyed.

The method of destroying the vitality of the dental pulp will not be considered in this paper. It makes no difference to me how the pulp is destroyed as long as the results are satisfactory. In my own practice I use both pressure anesthesia and devitalization. In cases of pressure anesthesia, where I know in advance that this operation is to be performed, I generally make one application of desensitizing paste which permits my reaching the organ without pain in those cases where there is a cavity and of making a depression or cavity painlessly in those cases where no cavity exists. In this manner the desired end is accomplished with very little pressure and the least pressure used the better the results will be. I also desire to state that in many cases I still use arsenic trioxid.

### ACTION OF ARSENIC TRIOXID

At the present time there is considerable discussion in dental literature as to whether or not the effects of arsenic trioxid when applied to a dental pulp are self-limiting. Some men of high standing in the profession are of the opinion that the drug is too destructive an agent to be used for the purpose of pulp devitalization. It seems to the author that these opinions are not well founded when we consider the long clinical record of the drug as a devitalizing agent. It has been used since 1836. There can be no question regarding the harmful effects of the drug when injudiciously employed. Its action is not self-limiting. Its effects are produced only after the drug is absorbed by the tissue elements, altering or destroying their vital processes in an obscure manner. The reason why the drug may be safely employed in devitalizing the pulp of a fully erupted tooth lies in the fact that here the effects are confined to the pulp tissue, since the organ dies by strangulation at the apex, due to the congestion caused by the drug when so used. Thrombosis and death of the pulp takes place and, owing to the small formina of the tooth root, absorption of the drug by continuity of tissue is prevented. In those cases where the apices of the roots are not fully formed, the drug, if used at all, should be left sealed in the tooth only a short time.

### DIAGNOSIS OF DEAD PULPS

It is not a difficult matter, as a rule, to differentiate between the teeth containing living or dead pulps. The clinical history, the color of the tooth, its response or non-response to thermal or electrical stimuli, will lead us to suspect that the pulp is dead or alive. Now we are ready to confirm our diagnosis. One of the best means of confirming the diagnosis of a dead pulp is the use of electricity by an instrument known as the "vibrating coil." This consists of a dry cell and a vibrating coil with a switch to control the current. The cord leading from the positive pole is arranged with a holder so that a broach may be attached; cotton is wrapped around the broach and moistened when it is applied to the suspected tooth. The cord from the negative pole is attached to a metal holder, which is held by the hand of the patient; thus the circuit is completed. A stronger current may be had by moving the positive pole to the right on the instrument board or by gradually withdrawing the cylinder which runs through the coil. One should always start with a weak current, gradually increasing the strength if no response is obtained. It is best to apply the current first to a tooth known to be vital, and use this as a control or guide. When two approximating teeth contain metal fillings, the current should be applied to the tooth so as not to come in contact with the filling of the adjoining tooth, which, if vital, would respond, leading one to believe that the tooth to which the current is applied is vital, when, as a matter of fact, it may be pulpless.

After the diagnosis of a dead pulp has been confirmed, the pulp chamber should be freely opened with a suitable round or special pulp chamber bur. It is not enough here to simply determine whether the pulp is dead or alive; but, if dead, an effort should be made to determine whether the condition is one of true gangrene with its characteristic odoriferous and gaseous end products or whether, as sometimes occurs, the active pyogenic microorganisms have liquefied a part or all of the bulbous portion of the pulp, forming drops of pus in the pulp chamber with practically no odor, and with the tissue in the canals still vital.

### TREATMENT OF PULPLESS TEETH

When a patient presents with a pulpless tooth, the original pulp tissue is generally either in a moist gangrenous condition, as a result of the action of bacteria, or a previous attempt has been made to remove the pulp and fill the canals. In either case, the first treatment here, in my opinion, should be a dressing of formocresol. Other preparations may be just as effective, but this is the one I have used for years and about which I can speak with assurance. Under aseptic precautions, this remedy should be placed in the pulp chamber only, and the dressing retained with cement. This dressing should remain at least twenty-four hours, and in the interim a radiograph of the tooth and periapical area should be obtained. I know that many good men today advocate the immediate cleansing of a gangrenous canal; but I am firmly of the belief that the safest and best practice is to first apply at least one dressing of formocresol. Gilmer, Moody, and others have found that the predominating organism in the conditions resulting from a gangrenous pulp, therefore the group active in bringing about the decomposition is a streptococcus.

One initial treatment of formocresol in these cases, while it may not kill all the organisms present, will certainly lessen the number and lower the virulency of those remaining. Surely no one will deny the favorable and valuable effects of formaldehyd gas on the intermediate and end products of pulp decomposition. With these poisonous and irritating products chemically neutralized; with the bacteria, inaugurating the process, killed or their virulency lowered, we may the more safely, I believe, work to the root end, clean and enlarge the canal by a chemico-mechanical process. By this I mean the use of chemicals in connection with broaches, reamers, files and other necessary instruments. At the present time, so far as I know, there are three chemical agents in general use for this purpose—metallic potassium and sodium, as advocated by Rhein; sulphuric acid by Callahan, and phenolsulphonic acid by myself. In this paper I shall only consider the use of acids.

### ADVANTAGES OF PHENOLSULPHONIC ACID

The chemical action of sulphuric and phenolsulphonic acids for the use under consideration is practically the same; and they will be discussed here conjointly. Personally, I prefer phenolsulphonic acid. Its consistency is thick and syrupy in the 80 per cent. strength which is recommended; thus a drop will adhere to the end of a broach or other applicator, permitting it to be carried even into the canals of upper teeth without difficulty. The acid does not materially effect a broach, and if any of it accidentally gets on the enamel of the crown of the tooth and is not noticed for a time, it does not decalcify and whiten the spot to the extent that sulphuric acid would do. It might be supposed by those who have not used phenolsulphonic acid that, possessing the above-mentioned advantages over plain sulphuric acid, it would be less effective in the canal. I have not found this to be the case. The agent has one possible disadvantage. In using either of these acids, after they have been introduced into the canal for a time and the walls thereof filed with a suitable broach, they should be neutralized with a solution of sodium bicarbonate. In the case of sulphuric acid the salt formed as a result of the reaction is sodium sulphate, a freely soluble salt. With phenolsulphonic acid the salt formed is sodium phenolsulphonate, which is not as soluble as sodium sulphate. I can conceive how it might be possible in a very fine canal to temporarily block it with the salt formed. An excess of water would readily dissolve the salt, or by using a solution of sodium bicarbonate of not more than 10 per cent. strength the salt would be dissolved as soon as formed by the reaction. This is a thing which probably would never occur in practice; but I mention it as a possible disadvantage.

### PULP PARTIALLY ALIVE

In those cases where the pulp tissue is gangrenous in one or more canals of a multirooted tooth and alive in the other one or two canals, as the case may be, we will find much satisfaction in using the formocresol remedy. These are exceptional cases, and it is difficult to know whether this condition exists until the second sitting. If there be much vitality in the live pulp tissue, the formaldehyd in the remedy will doubtless make the tooth ache, but after we know the condition our method of procedure is simple, and the results will be certain. A small pledget of cotton dipped in the remedy can gently be placed over the mouths of the canals which contain gangrenous material, and a thin, quick-setting cement flowed over the cotton. After the cement has set, the live pulp may generally be desensitized and removed with very little pain by gently working phenolsulphonic acid up or down the canal with a fine broach. Formerly these were difficult cases to treat, but with a remedy which can be hermetically sealed in a gangrenous canal, the procedure is materially simplified.

### PULPAL ABSCESS

It was previously mentioned that one may occasionally open in to a suspected tooth, having all the symptoms of a dead pulp, and find a drop of pus in the bulbous portion of the pulp chamber with the remaining portion still alive. Some writers call this condition a septic pulp or pulpal abscess in contradistinction to gangrene or true putrescence (presence of gases). In such a case, any remedy containing formaldehyd may cause the tooth to ache; therefore, this must be taken into consideration, and in the treatment of so-called septic pulps, the pus should be washed out of the cavity with a warm antiseptic solution, the cavity dried, and asepsis established by hermetically sealing in a much smaller amount of the formocresol than that generally used in cases of true gangrene. If this precaution is taken the tooth will not ache long, even in cases where no odor is present and the vital tissue is still quite sensitive. This remedy will sterilize the tissue, after which, at the second sitting, the pulp can be anesthetized or devitalized as usual.

### REMOVING OLD ROOT FILLING

In those cases where a previous attempt had been made to fill the canal with guttapercha, it is necessary, after the initial formocresol treatment, to first remove the old root filling. Generally in these cases there is only one cone in each canal, in which event the acid will soon work its way around the cone when it may be loosened with a suitable instrument and removed in one piece. However, occasionally it becomes necessary to remove a filling where the guttapercha has been firmly placed in the canal.

Here the use of xylol, or xylene, as it is also called, will aid materially in disintegrating the guttapercha. This is dimethylbenzene, and while the agent does not dissolve the guttapercha in the sense that chloroform would, it readily attacks and disintegrates the material, making its removal, after a few minutes, comparatively easy.

### REMOVING BLOCKADE IN CANAL

We should never use any drug or chemical blindly. Therefore, if in working up or down a canal, we find it blocked and are unable to reach the end, instead of going on blindly using only one chemical, whatever it may be, and running the risk of making a false pocket, we should stop and ascertain, if possible, and it is generally possible, the nature and character of the material causing the blockade. It may be a remnant of an old guttapercha cone; it may be pulp tissue undecomposed which has been inadvertently packed in the canal; it may be some inorganic material, such as a pulp nodule, secondary dentin, or other loosened inorganic material which has been pushed into the canal. If we have reasons for concluding that an old guttapercha filling is causing the blockade, xylol is the chemical to use; if we think it is organic material, the alkalies, like potassium and sodium or a strong solution of one of the hydroxids of these metals, is indicated; if inorganic material, sulphuric acid or phenolsulphonic acid will best answer our purpose.

Whenever we find these blockades, as we so frequently do where the canals have been treated before, and when we select and use the proper chemical and with a twist broach we slowly and carefully, but nevertheless surely, twist our way to the end of the root; and then when we fully appreciate that we are actually to the end—in language of Briggs, the cartoonist, "Oh! ain't that a grand and glorious feelin'?"

It is well understood, I trust, that all this work should be done under as nearly aseptic conditions as can be done in the modern dental office; and, in closing, I will say that no dental office is complete today without practical means of sterilizing all instruments and materials used by the four methods of dental sterilization, viz., direct flame, moist heat, dry heat, and chemical agents. The responsibility of having all instruments and materials used in dental surgery sterile, where it is possible to cause an infection through an open wound or otherwise, is rightfully thrown upon the dentist; and in assuming this responsibility, he should study carefully the various methods of sterilization and exercise judgment in his application of the principles employed; then when it is necessary to remove a pulp from whatever cause, it may be done without fear and trembling; and without following the highly questionable dictum that all pulpless teeth had better be extracted. In doing this work my advice is to continue to do your best, which, it must be admitted is very good.

